# Polymer Modified Carbon Fiber Microelectrodes for Precision Neurotransmitter Metabolite Measurements

**DOI:** 10.1149/1945-7111/abcb6d

**Published:** 2020-11-26

**Authors:** Pauline Wonnenberg, Whirang Cho, Favian Liu, Thomas Asrat, Alexander G. Zestos

**Affiliations:** 1 Department of Chemistry, American University, Washington, D.C. 20016, United States of America; 2 Center for Behavioral Neuroscience, American University, Washington, D.C. 20016, United States of America

## Abstract

Carbon fiber-microelectrodes (CFMEs) are considered to be one of the standard electrodes for neurotransmitter detection such as dopamine (DA). DA is physiologically important for many pharmacological and behavioral states, but is readily metabolized on a fast, subsecond timescale. Recently, DA metabolites such as 3-methoxytyramine (3-MT) and 3,4-dihydroxyphenylacetaldehyde (DOPAL) were found to be involved in physiological functions, such as movement control and progressive neuro degeneration. However, there is no current assay to detect and differentiate them from DA. In this study, we demonstrate the co-detection of similarly structured neurochemicals such as DA, 3-MT, and DOPAL. We accomplished this through electrodepositing CFMEs with polyethyleneimine (PEI) and poly(3,4-ethylenedioxythiophene) polystyrene sulfonate (PEDOT:PSS) polymers. This endowed the bare unmodified CFMEs with surface charge, physical, and chemical differences, which resulted in the improved sensitivity and selectivity of neurotransmitter detection. The differentiation and detection of 3-MT, DOPAL, and DA will potentially help further understand the important physiological roles that these dopaminergic metabolites play *in vivo*.

Dopamine (DA) is an important neurotransmitter associated with motor and cognitive functions such as movement, motivation, and reward. Its impairment is involved with several neurological processes and neuropsychiatric disorders, specifically Alzheimer’s disease, schi-zophrenia, drug abuse, and Parkinson’s disease.^1^ The ability to diagnose these diseases requires the development of a method with the ability for rapid neurotransmission detection in awake and behaving subjects. Meanwhile, metabolites of DA are formed through the oxidative catalyzation of the DA (Scheme 1). DA is enzymatically metabolized via catechol-O-methyl transferase (COMT), which replaces a hydroxyl group with a methoxy group to form 3-methoxytyramine (3-MT). COMT inhibitors are frequently used as treatments for Parkinson’s disease. On the other hand, 3,4-dihydroxyphenylacetaldehyde (DOPAL) is formed through the catalyzed oxidative deamination of the monoamine oxidase (MAO) enzyme, which replaces the amine group of DA with a carbonyl group.^2^ MAO inhibitors are commonly used as anti-depressants and anti-psychotic drugs, which has made their study of interest to many scientists. The detection of these enzymatic chemical events converting DA to 3MT and DOPAL is challenging, because the rate of metabolism is in the range of seconds.^3^

3-MT has been found to be physiologically important as the administration of 3-MT reverses the effects of dyskinesia in DA deficient Parkinsonian mice.^5–7^ On the other hand, DOPAL is a toxic metabolite of DA whose accumulation in the striatum can lead to the death of dopaminergic neurons in the striatum and hence cause Parkinson’s disease.^2^ The accumulation of DOPAL has recently been seen as a biomarker for Parkinsonian and pre-Parkinsonian patients.^2^ In addition, 3,4-dihydroxyphenylacetic acid (DOPAC) is a metabolite breakdown product of DA, where the amine group on the DA molecule is replaced by a carboxyl group, being modulated in response to a psychological stress and predators exposure, which can be seen in Scheme 2.^8–10^

Over the past thirty years, fast-scan cyclic voltammetry (FSCV) has attracted great attention for rapid detection of neurotransmitters.^11^ FSCV utilizes rapid potential sweeps to oxidize and reduce analytes of interest. In conventional techniques, such as microdialysis^12,13^ employing relatively large probes, are likely to cause tissue damage during in vivo implantation and lack the spatial and temporal resolution compared to FSCV measurements.^14–16^ In conjunction with FSCV, CFMEs have the capability to detect electrochemically active neurotransmitters (i.e., DA) based on the redox reaction and the resulting cyclic voltammogram (CV), which provides unique chemical fingerprints.^17^

DA is positively charged at a physiological pH (~7.4) in vivo, as the amine is protonated. The current assays of FSCV are optimized for DA detection. The inherent CFMEs are functionalized with negatively charged oxide, hydroxyl, and carboxylic acid functional groups that allow the adsorption of the cationic DA on the electrode surface.^18^ Moreover, the current waveform for FSCV testing is also optimized for DA detection. A holding potential of −0.4 V is applied that allows DA to adsorb onto the electrode surface.^19–21^ The negative holding potential allows the positively charged DA to preconcentrate and adsorb on the electrode surface. Upon scanning to 1.3 V at a scan rate of 400 V s^−1^, DA is oxidized to dopamine-ortho-quinone (DOQ) and then subsequently reduced to DA. The switching potential of 1.3 V etches the electrode surface to renew the electrode surface, increase the electroactive surface area (roughness), and functionalizes with negatively charged oxide groups.^22^ Despite DA being the most common target for FSCV, assays for other neurotransmitter metabolites have not been widely developed.

The challenge of electrochemical detection of neurotransmitters is that molecules having only slight differences in redox potential can interfere with each other and impair selectivity. In order to overcome this problem, more recently, electrode surface modifications have been used to enhance both the temporal and spatial resolution of CFMEs.^23–27^ Altering the surface chemical and physical properties of the electrode can enhance electrochemical sensitivity and the resulting improvement of sensitivity and selectivity of electrode.^28–31^ The Hashemi group has utilized Nafion coatings on carbon fiber microelectrodes to enhance serotonin detection,^32^ while Venton and colleagues have shown that CNT modified microelectrodes^33^ enhance both sensitivity and temporal resolution of neurochemical measurements.^23^ Moreover, CNT yarns,^34–36^ cationic or anionic polymers, such as polyethyleneimine (PEI),^37,38^ Nafion, poly(3,4-ethylenedioxythiophene) (PEDOT)-Nafion,^39–41^ and others have been used to enhance the detection ability by increasing electroactive surface area of CFMEs while reducing the surface fouling.^28^

In this study, polymer coatings were utilized to enhance the detection sensitivity of 3-MT and DOPAL as well as selectivity from DA. Specifically, PEI^38^ and PEDOT:PSS^41^ were used to functionalize the surface of the CFMEs to differentiate DA and its metabolites. PEI coatings provide a positive charge to the surface of CFMEs due to the protonated amine groups, which preferentially attract the negatively charged DOPAC and DOPAL. Conversely, PEDOT:PSS coatings onto the surface of the CFMEs preferentially attracts DA and 3-MT adsorption through electrostatic and altered surface properties of the modified electrode to create a superior electrode material. Overall, the co-detection, discrimination, and differentiation of DA, 3-MT, and DOPAL at a fast, subsecond temporal resolution will further help understand the important physiological roles of these neurotransmitter metabolites in vivo and the metabolic breakdown apart of DA to metabolites via MAO and COMT enzymes.

## Experimental: Methods and Materials

### Materials.—

The solutions were prepared according to the protocol found in previously reported literature.^28,42^ DA, DOPAC, 3-MT, DOPAL, polyethyleneimine (PEI), and 3,4-ethylenedioxythiophene (EDOT) were obtained from Sigma-Aldrich (St. Louis, MO). Poly (styrene sulfonic acid) sodium salt (PSS) was obtained from Alfa Aesar (Ward Hill, MA). A 10 mM stock solution of each anlyte was prepared with 0.1 M perchloric acid, then diluted to 1.0–100 *μ*M with artificial cerebral spinal fluid buffer (aCSF) (145 mM NaCl, 2.68 mM KCl, 1.40 mM CaCl_2_.2H_2_O, 1.01 mM MgSO_4_.7H_2_O, 15.5 mM Na_2_HPO_4_, and 0.45 mM NaH_2_PO_4_.H_2_O with the pH adjusted to 7.4). All the aqueous solutions were prepared with deionized water (Millipore, Billerica, MA). Epon 828 Epoxy was obtained from Miller-Stephenson (Morton Grove, IL) and diethylenetriamine hardener was obtained from Fisher Scientific (Waltham, MA).^28,42^

### Methods.—

The procedure for the construction of carbon fiber-microelectrodes was applied as previously reported.^28,42^ A single carbon fiber (7 *μ*m, Goodfellow, Huntingdon, England) was aspirated into a cylindrical glass capillary (1.2 mm by 0.68 mm, A-M Systems, Inc., Carlsborg, WA) using a vacuum pump (DOA-P704-AA, GAST, Benton Harbor, MI).^43^ Subsequently, carbon fibers were pulled using a vertical capillary puller (Narishige, model PC-100 and PE-22, Tokyo, Japan) and the fiber was cut to length of approximately 100–150 microns. Glass insulated electrodes were epoxied with Epon 828 epoxy (Miller-Stephenson, Morton Grove, IL) and diethylenetriamine (Sigma Aldrich, Milwaukee, WI). Protruding carbon-fiber microelectrode tips were dipped in the epoxy hardener mixture for approximately 15 s and then rinsed in acetone to remove residual epoxy hardener. The electrodes were cured for 4 h at 125 °C oven.^28^ First, the electrodeposition of polyethyleneimine (PEI) polymer solution onto the surface of carbon-fiber microelectrodes (CFMEs) was performed as previously described.^28,42^ Linear PEI polymer (Mn ~60,000, Sigma Aldrich, Milwaukee, WI) was dissolved in methanol to make a 20 wt% solution. A triangle waveform was applied to the electrode from a holding potential of +1.5 V to −0.8 V and back at a scan rate of 100 mV s^−1^ and a frequency of 10 Hz for 5 min.^28^ Subsequently, 3,4-ethylenedioxythiophene:polystyrene sulfonate (EDOT:PSS) polymer solution was then electopolymerized onto the surface of abovementioned PEI-coated CFMEs to fabricate PEDOT-PEI coated CFMEs under the same triangle waveform settings. 10 mM EDOT:PSS (Sigma-Aldrich, St. Louis, MO) was dissolved in 1 wt% aqueous solution of PSS.

For the bare and polymer coating comparisons, 5 *μ*M DA, 5 *μ*M 3-MT, and 20 *μ*M DOPAL solutions were prepared in ACSF from the 10 mM stock solutions. Each ratio solutions were prepared using the aforementioned dilutions. The 1:1 DA to 3-MT solution was prepared by diluting each compound to a concentration of 2.5 *μ*M (total volume: 2 ml). The 1:10 DA to 3-MT solution was prepared by diluting each compound to a concentration of 0.5 *μ*M and 5.0 *μ*M, respectively (total volume: 2.2 ml). The 1:50 and 1:100 dilutions of DA to 3-MT and DA to DOPAL were prepared similarly in order to maintain a similar concentration of the metabolites of DA (3-MT and DOPAL) while lowering the concentration of DA enabling stronger detection of its metabolites. In addition, the 10:1 and the 100:1 dilution were prepared using a similar dilution, utilizing a fixed DA concentration and lowering the concentration of the DA metabolites.

Data was collected with the WaveNeuro FSCV system and a 5 MΩ headstage (Pine Instruments, Durham, NC, USA).^28^ Data then was analyzed using the HDCV software (University of North Carolina Chapel Hill, Mark Wightman) and a computer interface board (National Instruments PC1e-6363, Austin, TX, USA).^28^ The triangular waveform was used; a holding potential of −0.4 V to +1.3 V at a scan rate of 400 V s^−1^ and a frequency of 10 Hz. Both the scan rates (50–1000 V s^−1^) and concentrations (100 nM to 100 *μ*M) were varied from lower to higher values.^28^ A silver-silver chloride electrode was used as a reference to measure the potential of the CFME. Samples were tested in a flow injection analysis system (In Vitro/FSCV Microelectrode Flow Cell with xyz micromanipulator Translational Stage, Pine Instruments, Durham, NC).^28^ Buffer and samples were pumped through the flow cell using a NE-300 Just Infusion^TM^ Syringe Pump (New Era Pump Systems, Farmingdale, NY).^28^ The electrodes were equilibrated at the applied waveform for 10 min to prevent electrode drift between each run.^28^ All data was background sub-tracted to remove any non-faradaic currents.^28^

The bare CFMEs are back filled with a saturated solution of potassium chloride (KCl) and inserted in the flow cell system using the electrode holder and the proper headstage. The neurotransmitters (DA, DOPAC, 3-MT, and DOPAL) of interest are then injected, one by one, at a 0.1 ml quantity for the desired concentration, ranging from 1 *μ*M to 5 *μ*M. The same bare CFMEs are then electrodeposited with PEI and PEDOT and re-tested with the same neurotransmitters at the same concentrations to compare the effects of the polymer coating onto the carbon fiber.

SEM images were obtained with a JEOL JSM-IT100 (JEOL, Tokyo, Japan). Bare or polymer modified carbon fiber microelectrodes were sputter-coated with gold in Denton Desk II sputter coater. They were then placed onto conductive tape, which was then inserted into the SEM stage. The working distance was set to 10 mm and slightly adjusted to obtain optimal resolution and magnification, while the accelerating voltage ranged from 5 kV to 20 kV. Furthermore, the same JEOL software was also used to perform Energy-dispersive X-ray spectroscopy (EDS/EDX) measurements for chemical identification of polymers on the surface of the carbon-fiber microelectrode.

All data analysis was performed using GraphPad Prism 8. All error bars are standard error of the mean (SEM) unless otherwise noted.

## Results and Discussion

**Scheme 3.**
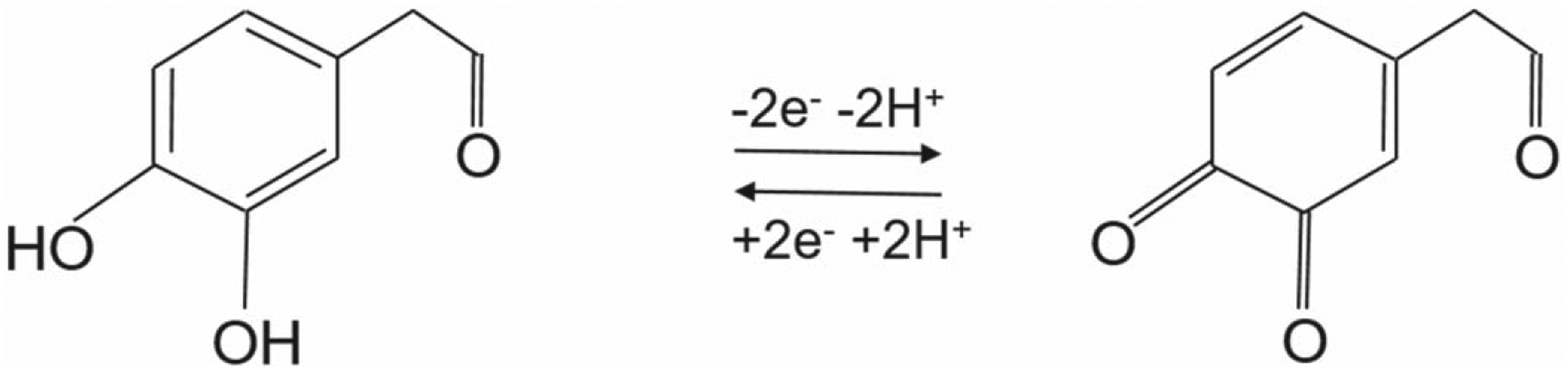
Electrochemical Oxidation of 3,4-dihydroxy-benzeneacetaldehyde (DOPAL).

**Scheme 4.**
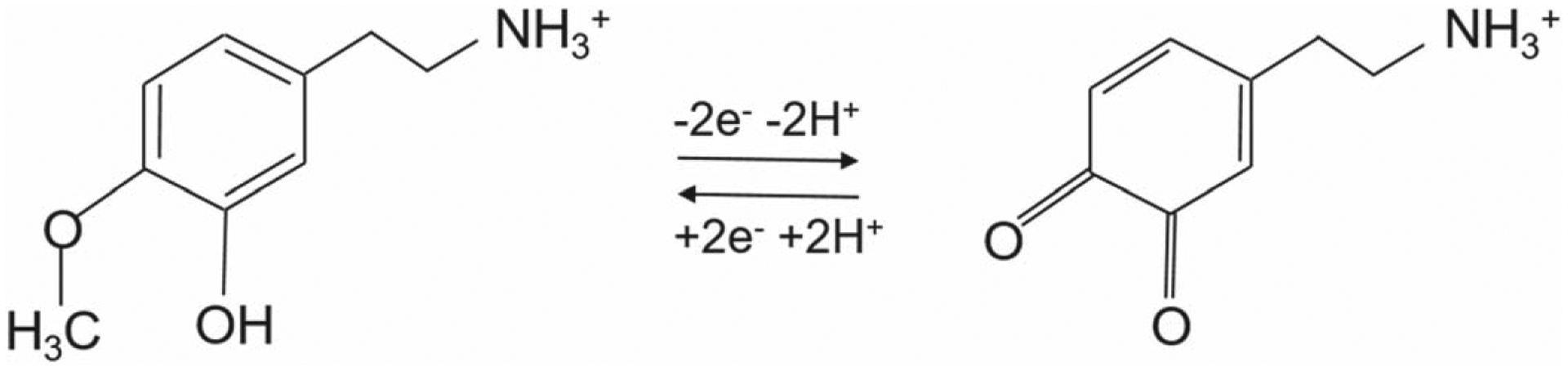
Electrochemical Oxidation of 3-Methoxytyramine (3-MT).

The electrochemical oxidation of 3-MT and DOPAL has been proposed to involve two-electron oxidation. Since DOPAL is a catechol compound, its redox mechanism is considered as similar to that of DA and DOPAC as shown in Scheme 3 below.^28^ It is an aldehyde as opposed to an amine (DA) and carboxylic acid (DOPAC). Therefore, it undergoes a two-electron reversible oxidation similar to that of DA. On the other hand, the oxidation mechanism of 3-MT is not as straightforward due to the presence of the methoxy group as a substitute for hydroxyl moiety, which, unlike DA or norepinephrine, is not a catechol or catecholamine. Previous studies depicting the electrochemical oxidation of catechol and 4-hydroxy-3-methoxy-phenyl compounds via chemiluminescence detection hypothesized that methoxy is oxidized into a quinone.^44^ Therefore, we hypothesize that 3-MT is also oxidized into a quinone as well upon electrochemical oxidation through the application of a positive voltage (Scheme 4).

**Figure 1.**
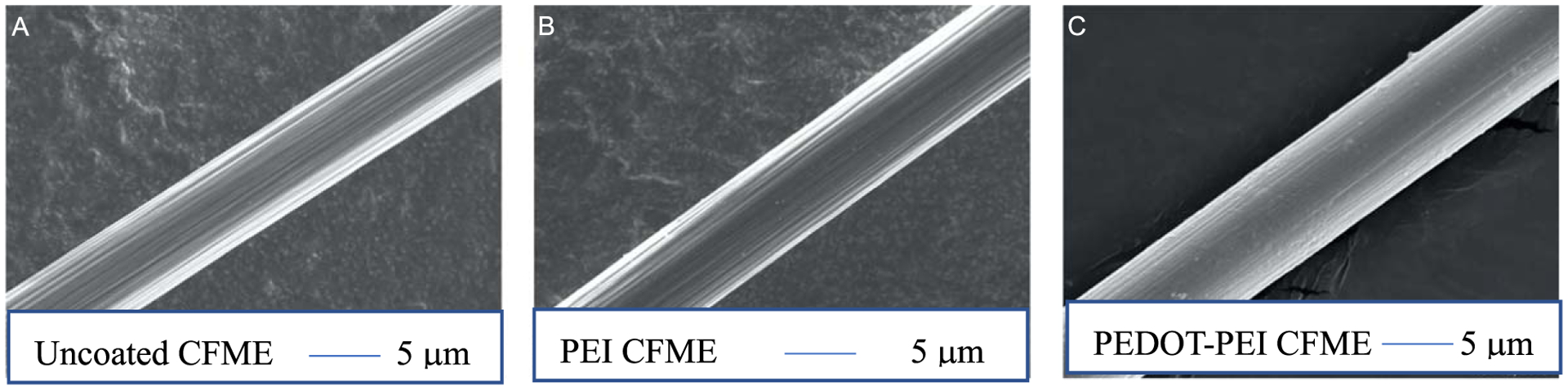
SEM images of (A) uncoated CFMEs approximately 7 *μ*m in diameter. (B) CFMEs electrodeposited with polyethyleneimine (PEI). A thin layer of polymer evenly coats the surface of the electrode and fills the ridge of the fiber. (C) CFMEs electrodeposited with poly(3,4-ethylenedioxythiophene)(PEDOT)-PEI. A thin layer of polymer coats the surface of the electrode and fills the ridge of the fiber to a greater extent than the PEI-coated carbon fiber (B).

As shown in the scanning electron microscope (SEM) images (Fig. 1A), the bare carbon fiber has deep grooves and ridges as they are thermally spun from polyacrylonitrile (PAN) and pyrolyzed at temperatures about 1000 K.^28,45,46^ The diameter of bare carbon fibers is approximately 7 *μ*m. PEI was electrodeposited onto the surface of CFMEs as shown in Fig. 1B. Since PEI is composed of repeating units of amine groups (pKa ~ 10), coating this polymer onto carbon fibers provides a positive charge at a physiological pH of 7.4 in aCSF buffer.^28^ Subsequently, PEDOT:PSS was electrodeposited onto the surface of PEI coated CFMEs. SEM images were taken to confirm a complete and thorough coating of the PEDOT-PEI polymer. A thin and uniform coating of polymer on the surface of the microelectrode surface enhances electron transfer and neurotransmitter adsorption. Moreover, the presence of sulfur and nitrogen on the surface of the polymer coated CFMEs confirmed that both PEDOT and PEI were successfully coated on the surface of the CFMEs as shown in Energy-dispersive X-ray spectroscopy (EDS/EDX) chemical analysis (Figs. S1A–S1B is available online at stacks.iop.org/JES/167/167507/mmedia). Nitrogen was not observed on the EDS data for bare, unmodified and PEDOT:Nafion modified CFMES (Figs. S1C–S1D)

## Sensitivity Comparison (Bare, PEI and PEDOT-PEI CFMEs)

**Figure 2.**
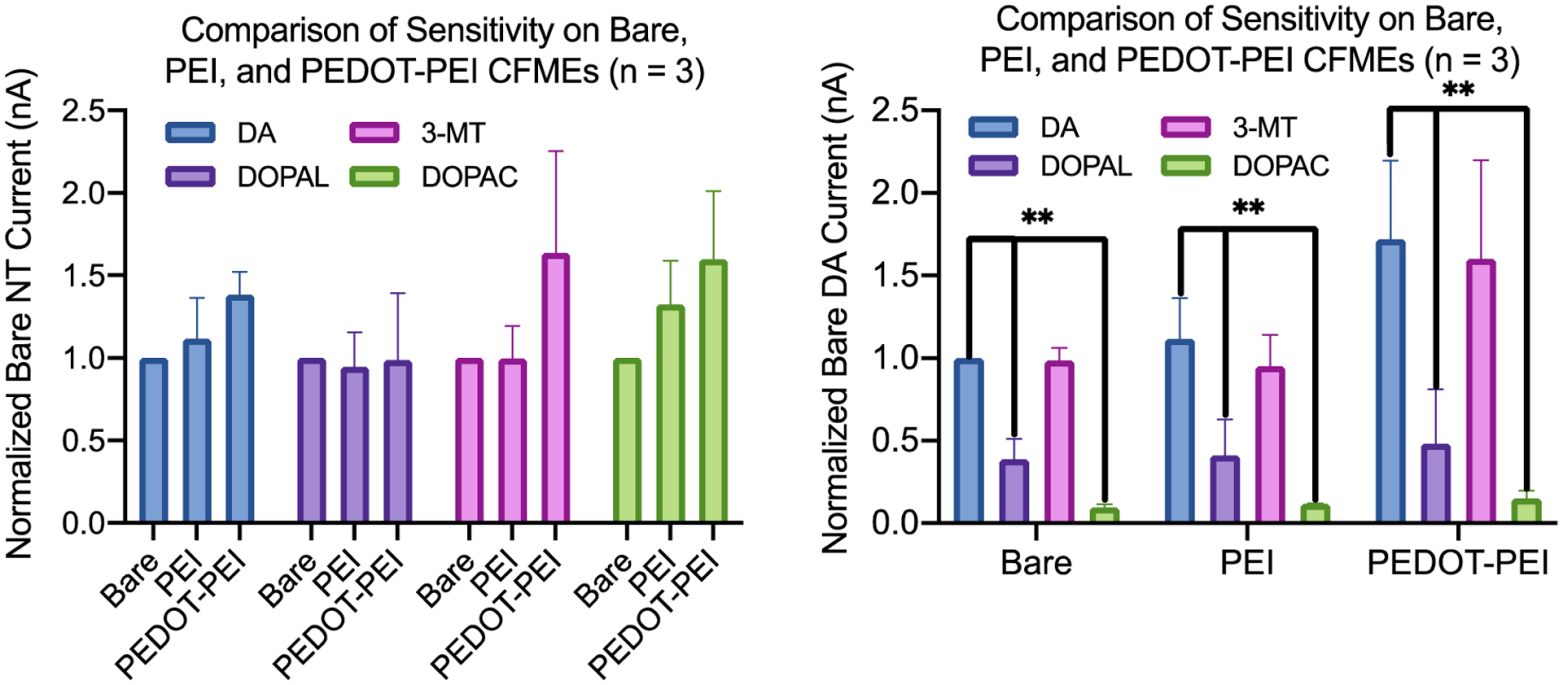
Effect of PEI and PEDOT-PEI polymer coatings on the detection sensitivity of dopamine (DA), 3-methoxytyramine (3-MT), 3,4-Dihydroxyphenylacetaldehyde (DOPAL), and 3,4-Dihydroxyphenylacetic acid (DOPAC). The currents were normalized for each neurotransmitter average current (n = 3). PEI coated CFMEs enhance the sensitivity of DA and DOPAC, but not 3-MT and DOPAL. PEDOT-PEI coated CFMEs enhance the sensitivity of DA, 3-MT, and DOPAC, but not DOPAL.

As shown in Fig. 2, the effect of polymer coatings on the detection sensitivity was studied for DA and its three metabolites (i.e., 3-MT, DOPAL, and DOPAC). The concentrations of the neurotransmitters tested were comparable to the levels found physiologically *in vivo*.^30,32^ The effect of PEI coatings appears to be most significant on the detection sensitivity of DOPAC (~30% increase) compared to other analytes. The introduction of PEI leads to a positively charged electrode surface that electrostatically attracted the anionic DOPAC to allow for the preconcentration and adsorption to the electrode surface, and hence, enhanced sensitivity and detection. Meanwhile, PEDOT-PEI polymer coatings significantly enhanced the detection sensitivity of both 3-MT and DOPAC (~60%) as well as DA (~40%), while having little effect on DOPAL sensitivity. This is explained by the altered surface properties of the PEI and PEDOT polymers, which increased the electroactive surface area of the CFMEs to enhance the detection of the biomolecules. Moreover, the lone pair from the amine (nitrogen) of the PEI was found to physiosorb to the wall of the graphene sheet of carbon electrode to promote an intermolecular charge transfer between the nitrogen and the carbon to enhance conductivity and hence detection, significantly.^47^ The raw data for the effect of PEI and PEDOT-PEI polymer coatings on the CFMEs sensitivity is shown in Fig. S3.

## Adsorption Control Experiments with 3-MT (Bare and PEI CFMEs)

**Figure 3.**
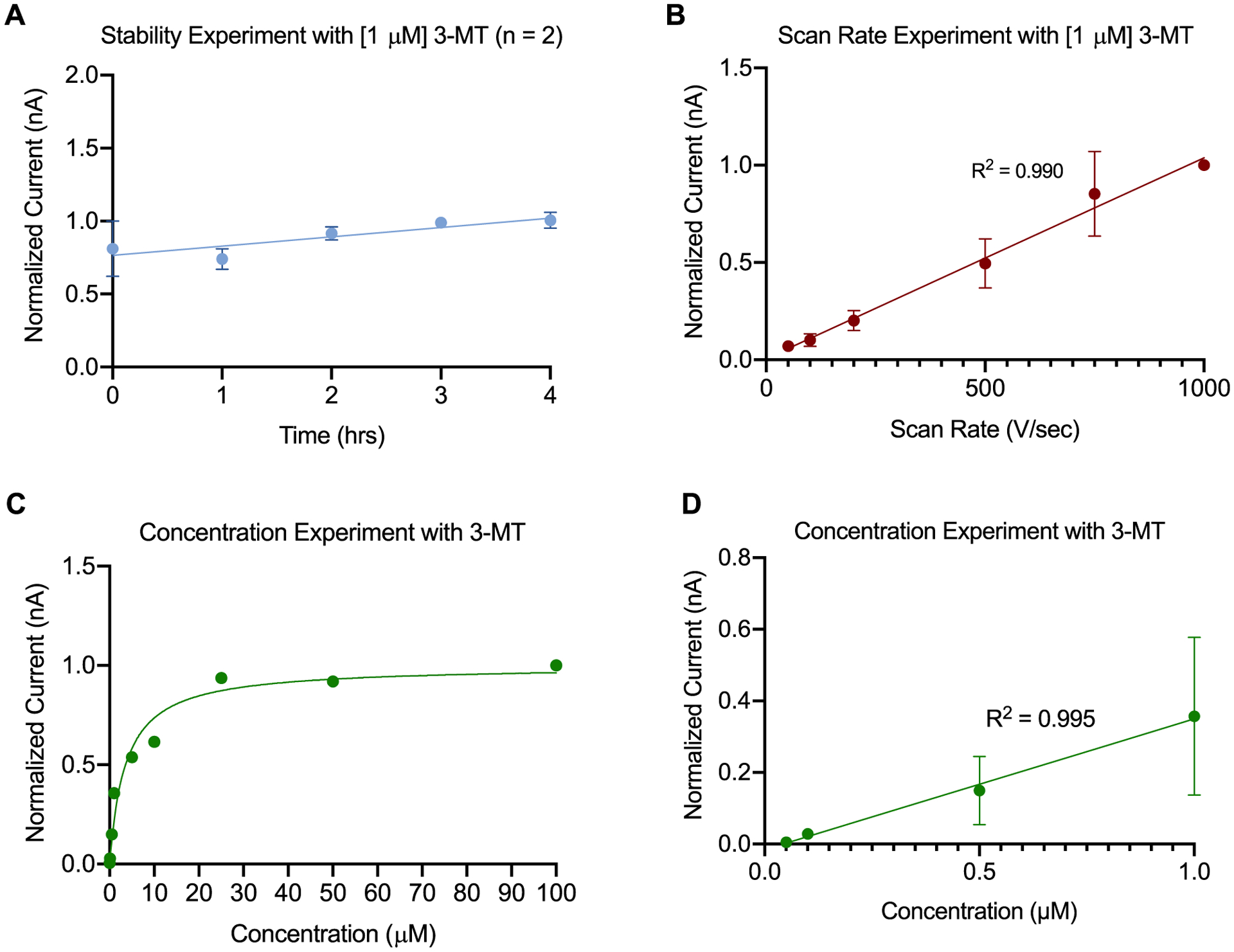
Adsorption control testing of CFMEs. (A) The electrode displays a stability towards 3-methoxytyramine (3-MT) detection (peak oxidative current) for at least 4 h. (n = 3). (B) Adsorption control testing for 3-MT (1 *μ*M) showing linear relationship between peak oxidative current and scan rate (50–1000 V s^−1^). R^2^ = 0.99 (n = 7). (C) The peak oxidative currents of the cyclic voltammograms (CV) for 3-MT showing asymptotic curve with respect to the concentration. R^2^ = 0.966 (n = 3). (D) Concentration experiment showing a linear relationship between 3-MT concentration (from 100 nM to 1 *μ*M) and peak oxidative current (R^2^ = 0.995, n = 3).

Several experiments including stability, scan rate, and concentration testings were performed to determine whether adsorption-controlled or diffusion controlled of the surface of bare CFMEs regarding the detection of 3-MT, thus demonstrating its utility for enhanced neurochemical detection. As demonstrated in Fig. 3A, bare CFMEs tend to be stable over the course of four hours. This experiment was performed to illustrate the integrity of the PEDOT-PEI polymer and to show that it is not leached or compromised from the surface of the electrode upon applying a waveform for up to four hours, which is the typical duration of an in vivo experiment. Figure 3B showed a normalized peak oxidative current of 1 *μ*M 3-MT towards bare CFMEs with respect to the scan rates in a range from 50 V s^−1^ to 1000 V s^−1^ (n = 7), which showed a linear relationship between scan rates and peak oxidative current. The linear relationship between peak oxidative current of 3-MT and scan rate shows that 3-MT is adsorption controlled (as opposed to diffusion controlled) to the surface of the bare CFME (R^2^ = 0.99). Moreover, an asymptotic curve was observed at higher concentrations of 3-MT, indicating that concentrations higher than 1 *μ*M are saturated and all adsorption sites are occupied by excessive 3-MT as shown in Fig. 3C (R^2^ = 0.966). In Fig. 3D, we observe that peak oxidative current was linear with respect to 3-MT concentration from 100 nM to 1 *μ*M (R^2^ = 0.995). The stability, scan rate, and concentration experiments for DA also showed that DA is adsorption controlled to the surface of both bare and PEI modified CFMEs (Figs. S2A–S2D). The absolute currents of the 3-MT adsorption control experiments are shown in Fig. S4.

## Adsorption Control Experiment with DOPAL (Bare CFMEs)

**Figure 4.**
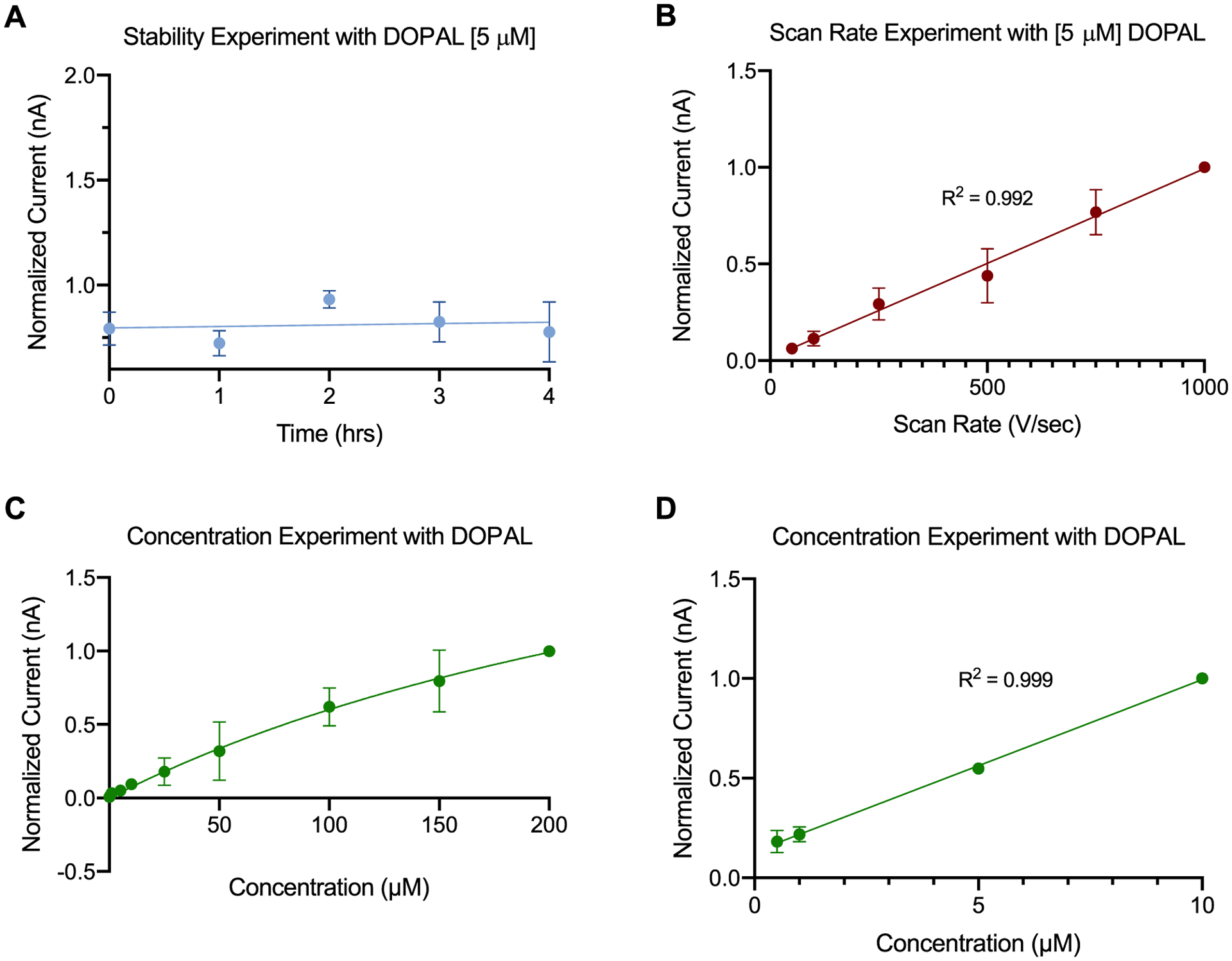
Adsorption control testing of CFMEs. (A) The electrode displays a stability towards DOPAL detection (peak oxidative current) for at least 4 h (n = 3). (B) Adsorption control testing for DOPAL (5 *μ*M) showing a linear relationship between scan rate (50–1000 V s^−1^) and peak oxidative current (R^2^ = 0.992, n = 3). (C) The peak oxidative currents for the cyclic voltammograms (CV) for DOPAL with respect to the concentration (R^2^ = 0.932, n = 3). (D) Concentration experiment showing a linear relationship between DOPAL concentration (100 nM to 10 *μ*M) and peak oxidative current (R^2^ = 0.999, n = 4).

Furthermore, we performed stability, scan rate, and concentration testings to determine whether DOPAL is adsorption-controlled or diffusion controlled for the surface of bare CFMEs, thus verifying its utility for enhanced neurochemical detection. Figure 4A demonstrates the stability of bare CFMEs over the course of 4 h for the detection of 5 *μ*M DOPAL. Figure 4B showed a linear relationship between normalized peak oxidative current of DOPAL and scan rates from 50 V s^−1^ to 1000 V s^−1^ (n = 3), confirming its adsorption-controlled interaction with the surface of the CFMEs (R^2^ = 0.992).^48^ Moreover, an asymptotic curve is found at higher concentrations, indicating DOPAL concentrations higher than 10 *μ*M are saturated and all adsorption sites are occupied by excessive DOPAL as shown in Fig. 4C (R^2^ = 0.932). Figure 4D provides the linear range for DOPAL detection and shows that peak oxidative current is linear with respect to 3-MT concentration from 100 nM to 10 *μ*M (R^2^ = 0.999). The DOPAL raw currents for the adsorption control experiments are included in Fig. S5.

**Figure 5.**
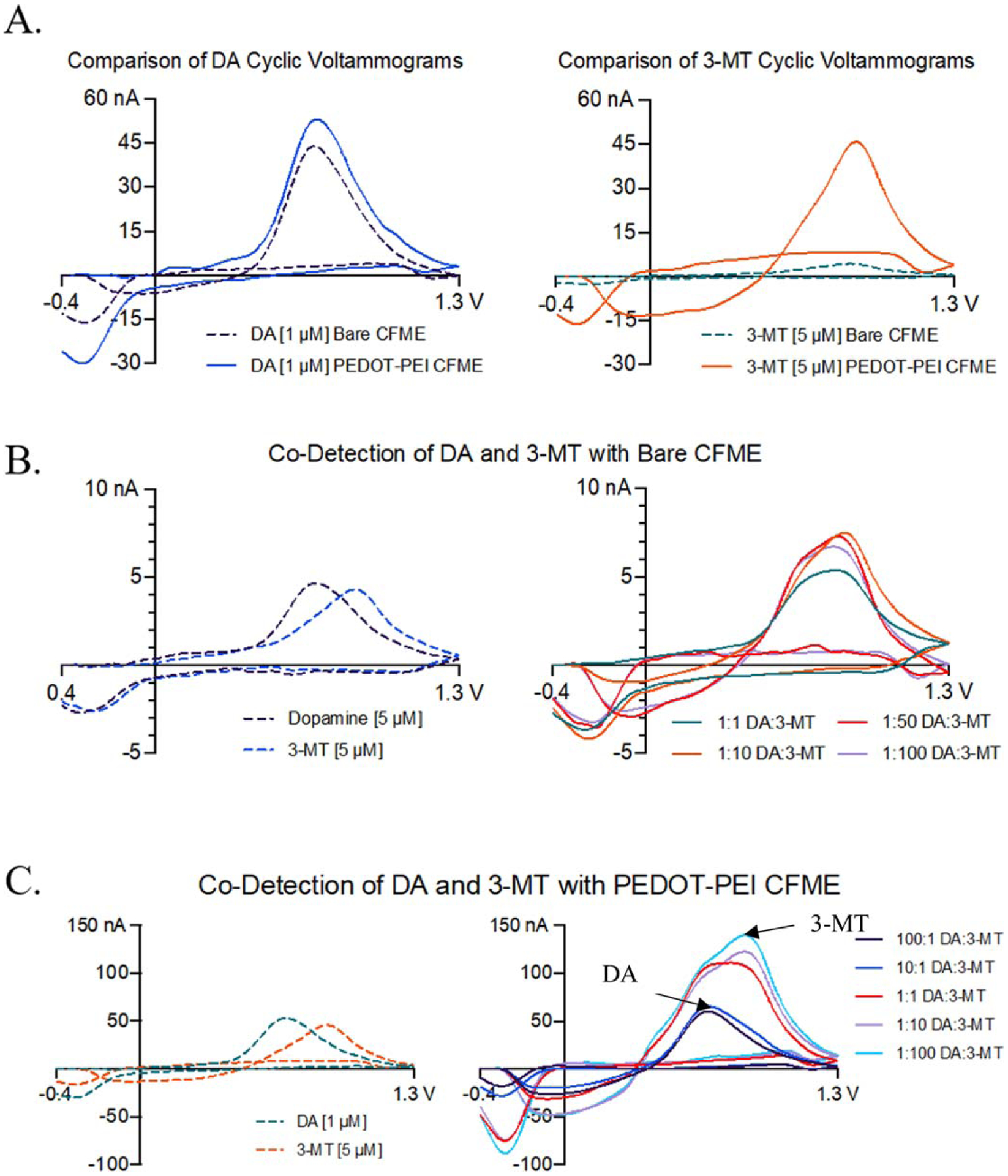
(A) Detection of dopamine (DA) and 3-methoxytyramine (3-MT) using a bare and PEDOT-PEI polymer coated electrode with a DA waveform. The dashed-lines represent the detection of DA and 3-MT with a bare CFME, whereas the solid lines represent that of PEDOT-PEI coated CFMEs. (B) Co-detection of DA and 3-MT with bare CFMEs, (C) Co-detection of DA and 3-MT with PEDOT-PEI coated CFMEs. These ratios correspond to the concentrations listed in Table SI.

The combination of the DA waveform and PEDOT-PEI polymer coatings onto the surface of CFMEs were used to co-detect mixtures of DA and 3-MT. As shown in Fig. 5, bare and PEDOT-PEI electrodeposited CFMEs were used to detect DA and 3-MT separately. Furthermore, concentration ratio mixtures of DA and 3-MT were analyzed to determine the co-detection enhancement with electrodeposited PEDOT-PEI polymer coatings. The analytes were differentiated and co-detected based on the position and shape of the respective cyclic voltammograms, which is a chemical fingerprint for biomolecule detection.

## Co-Detection of Dopamine and 3-MT (Bare and PEDOT-PEI CFMEs)

Figure 5A shows the enhancement of 1 *μ*M DA and 5 *μ*M 3-MT detection with PEDOT-PEI coated CFMEs compared to bare CFMEs. The peak oxidative current of DA increased from 43.90 nA to 53.06 nA, demonstrating the ability of PEDOT-PEI to enhance the detection of DA. It is noted that peak oxidative currents increased from 4.28 nA to 45.01 nA for 3-MT detection after PEDOT-PEI electrodeposition onto CFMEs, which displayed an over ten-fold increase in sensitivity for 3-MT detection. This significant increase in peak oxidative current indicates that 3-MT is more sensitive to PEDOT-PEI CFMEs than DA due to the increased electroactive surface area and altered physicochemical properties of the modified electrode surface. We measured different relative ratio combinations of 3-MT to DA, such as 1:1, 10:1, and 100:1 ratios, using FSCV since DA is generally more sensitive toward bare CFMEs than 3-MT. The relative concentrations for the aforementioned solutions were prepared by maintaining the concentration of DA fairly constant while lowering the 3-MT concentration. This enabled an enhanced selectivity to the bare and modified CFMEs for 3-MT detection (Table SI). As can be seen in Fig. 5B, peak oxidative current increases as the ratio of 3-MT to DA increases. We note that as the 3-MT concentration increases, the peak oxidative current potential of 3-MT can be discerned from DA as it is slightly shifted to a higher voltage compared to that of 1:1 between 3-MT and DA (an approximately 0.2 V difference) due to the slower electron transfer kinetics of 3-MT at CFMEs.^49^ Subsequently, the same experiment was repeated with higher relative concentration ratios of DA vs 3-MT in addition to the aforementioned ratios (100:1 and 1:10), on PEDOT-PEI CFMEs to demonstrate co-detection improvement compared to a bare CFME as shown in Fig. 5C. At 5 *μ*M, 3-MT can easily be differentiated from DA at 1 *μ*M because of the shift of peak oxidative current potential around 0.85 V, which is higher than that of bare CFMEs. In addition, their combination continued to have an additive behavior when they are co-detected as seen from 1:1, 1:10, and 1:100 ratios. Upon lowering the concentration of 3-MT while increasing concentration of DA (10:1, 100:1), the peak oxidative current of combined analytes becomes similar to that of just DA.

## Co-Detection of Dopamine and DOPAL (Bare and PEDOT-PEI CFMEs)

**Figure 6.**
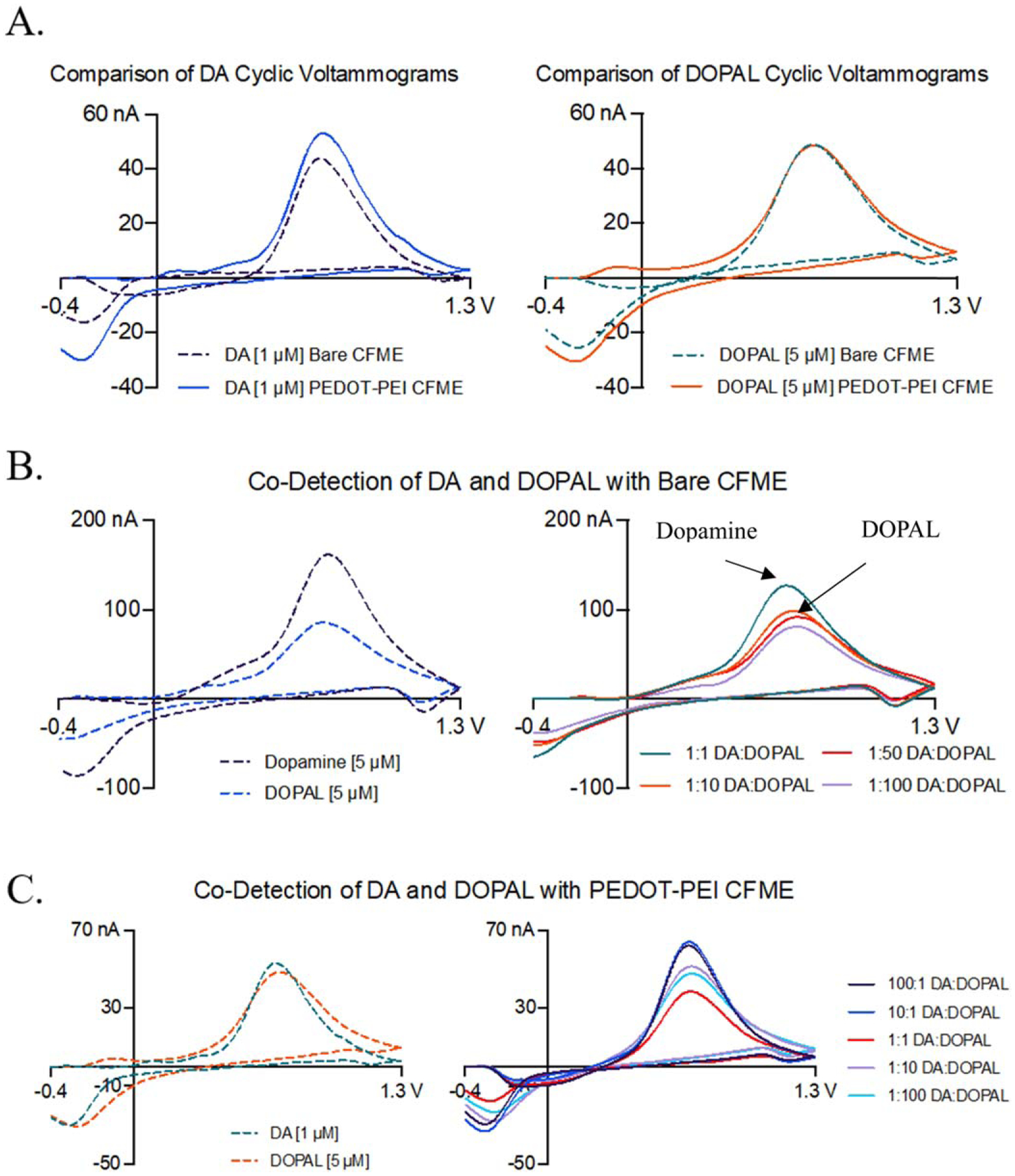
(A). Detection of dopamine (DA) and DOPAL using a bare and PEDOT-PEI polymer coated electrode with the DA waveform. The cyclic voltammograms (CV) of a solution of 1 *μ*M dopamine and 5 *μ*M DOPAL, respectively, are displayed with the same electrode before and after electrodeposition of PEDOT-PEI onto the CFMEs. (B) Co-detection of DA and DOPAL with bare CFMEs, (C) Co-detection of DA and DOPAL with PEDOT-PEI coated CFMEs. These ratios correspond to the concentrations listed in Table SI.

Lastly, the combination of the DA waveform and PEDOT-PEI polymer coatings onto the surface of CFMEs were used to co-detect DA and DOPAL as shown in Fig. 6A. Bare and PEDOT-PEI electrodeposited CFMEs were tested for both DA and DOPAL as well as for the co-detection of DA and DOPAL simultaneously upon altering the ratios of each analyte. They were also compared to determine the co-detection enhancement of PEDOT-PEI polymer coated CFMEs for both DA and DOPAL detection. DOPAL was tested at higher concentrations than DA since DA is more sensitive at CFMEs than DOPAL is. For the co-detection, different relative ratio combinations, such as 1:1, 1:10, 1:50, and 1:100, were performed on bare CFMEs. We kept the concentration of DOPAL constant while lowering the DA concentration to account for DA’s higher sensitivity at CFMEs than DOPAL (Table SI). As can be seen in Fig. 6B, the higher the ratio of DOPAL compared to DA was used, the lower peak oxidative current was observed. It appears that the oxidative peak potential is shifted by approximately 0.1 V with increasing the amount of DOPAL. The same experiment was repeated by using higher relative concentration ratios of DA to DOPAL in addition to the aforementioned ratios (100:1 and 1:10), on a PEDOT-PEI coated CFME to evaluate the co-detection improvement compared to bare CFMEs as shown in Fig. 6C. However, it shows a minimal difference between the peak oxidative potential of DOPAL and DA. It can be attributed to a slower electron transfer kinetics of DOPAL at the surface of the electrode with respect to DA. As the concentration of DA is higher than DOPAL, starting from 10:1 and higher, it is not possible to differentiate DA from DOPAL due to the similarity of chemical structure between DA and DOPAL compared to that of 3-MT.

## Conclusions

We have shown that the electrodeposition of PEDOT: PEI coatings onto CFMEs enhances the detection and differentiation of DA and dopaminergic metabolites such as 3-MT whereas having little effect on DOPAL. The altered physicochemical surface features of the polymer modified microelectrodes allowed differential adsorption of the metabolites to the surface of the electrode. This enabled the enhanced selectivity, sensitivity, and the co-detection and discrimination of DA and 3-MT metabolites from one another at the surface of the electrode, which was previously not possible with bare unmodified microelectrodes. On the other hand, the similarity in chemical structure between DA and DOPAL obscures the differentiation of oxidation potentials. This work is potentially important in differentiating and co-detecting these metabolites, which will be critical in understanding the physiological role of these metabolites apart from DA. It will provide vital information on the breakdown of DA to metabolites via MAO, COMT, and the role of these metabolites *in vivo*. Furthermore, *in vivo* investigations of the neurotransmitter pathways in zebrafish and rodents using polymer modified microelectrodes could help model human diseases or neurological and psychiatric disorders.

## Supplementary Material

Supplemental Data

## Figures and Tables

**Scheme 1.**
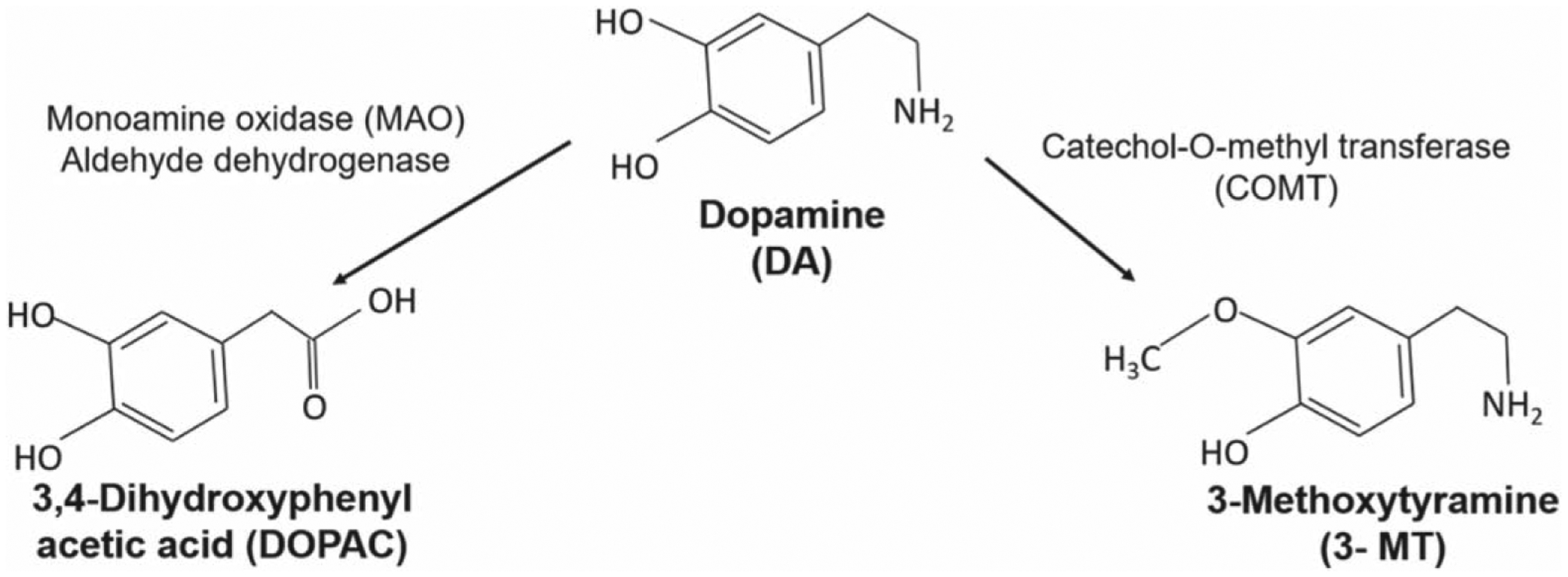
The enzymatic breakdown of DA to its inactive metabolites is carried out by catechol-O-methyl transferase (COMT) and monoamine oxidase (MAO).^4^

**Scheme 2.**
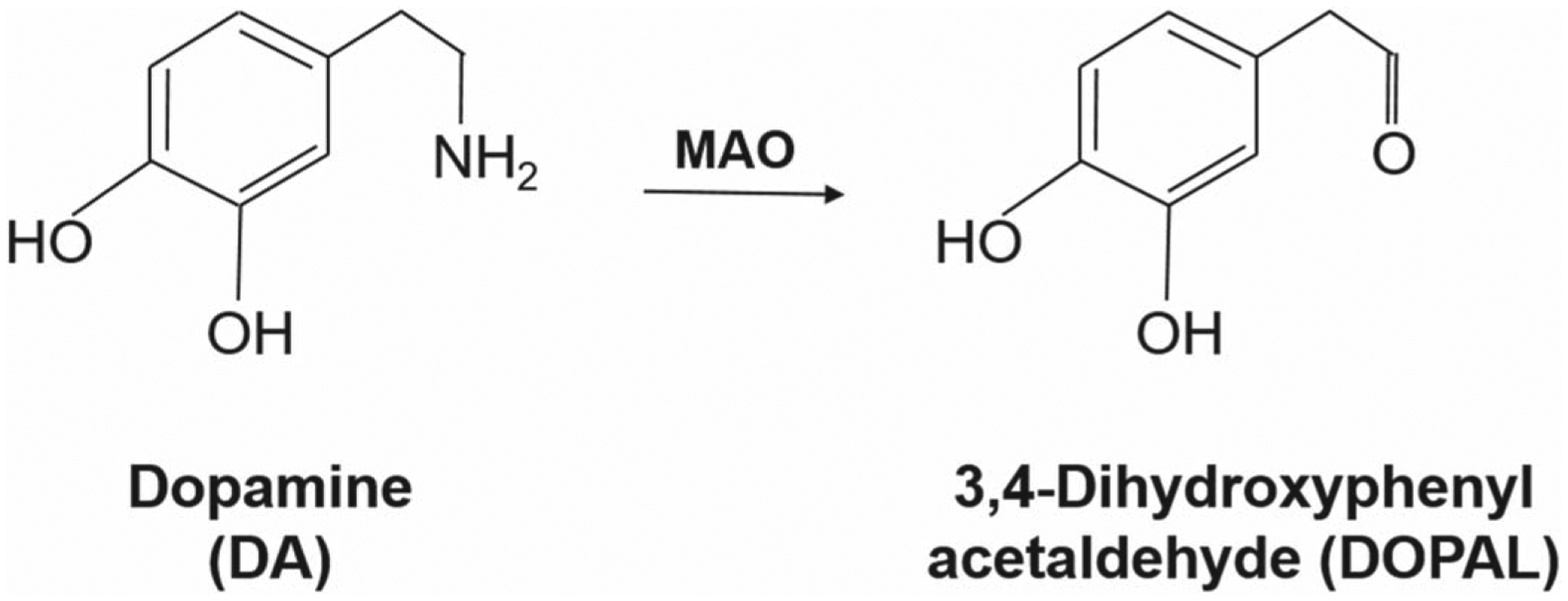
Metabolism of DA involving monoamine oxidase-catalyzed oxidative deamination to DOPAL.
