# Supplementary material for: Polymer Modified Carbon Fiber Microelectrodes for Precision Neurotransmitter Metabolite Measurements
Source: J Electrochem Soc. Author manuscript; Available in PMC 2021 Apr 28. (PMC8081299; doi:10.1149/1945-7111/abcb6d)
Supplement: Supplemental Data [file NIHMS1693720-supplement-Supplemental_Data.pdf]

Supporting Information

Polymer Modified Carbon Fiber Microelectrodes for Precision Neurotransmitter Metabolite Measurements

Pauline Wonnenberg[1], Whirang Cho[1], Favian Liu[1], Thomas Asrat[1], and Alexander G. Zestos[1,2,z]

[1]Department of Chemistry, American University, Washington, D.C. 20016, USA

[2]Center for Behavioral Neuroscience, American University, Washington, D.C. 20016, USA

[z]Author to whom correspondence should be addressed.
[z]*E-mail address*: zestos@american.edu.

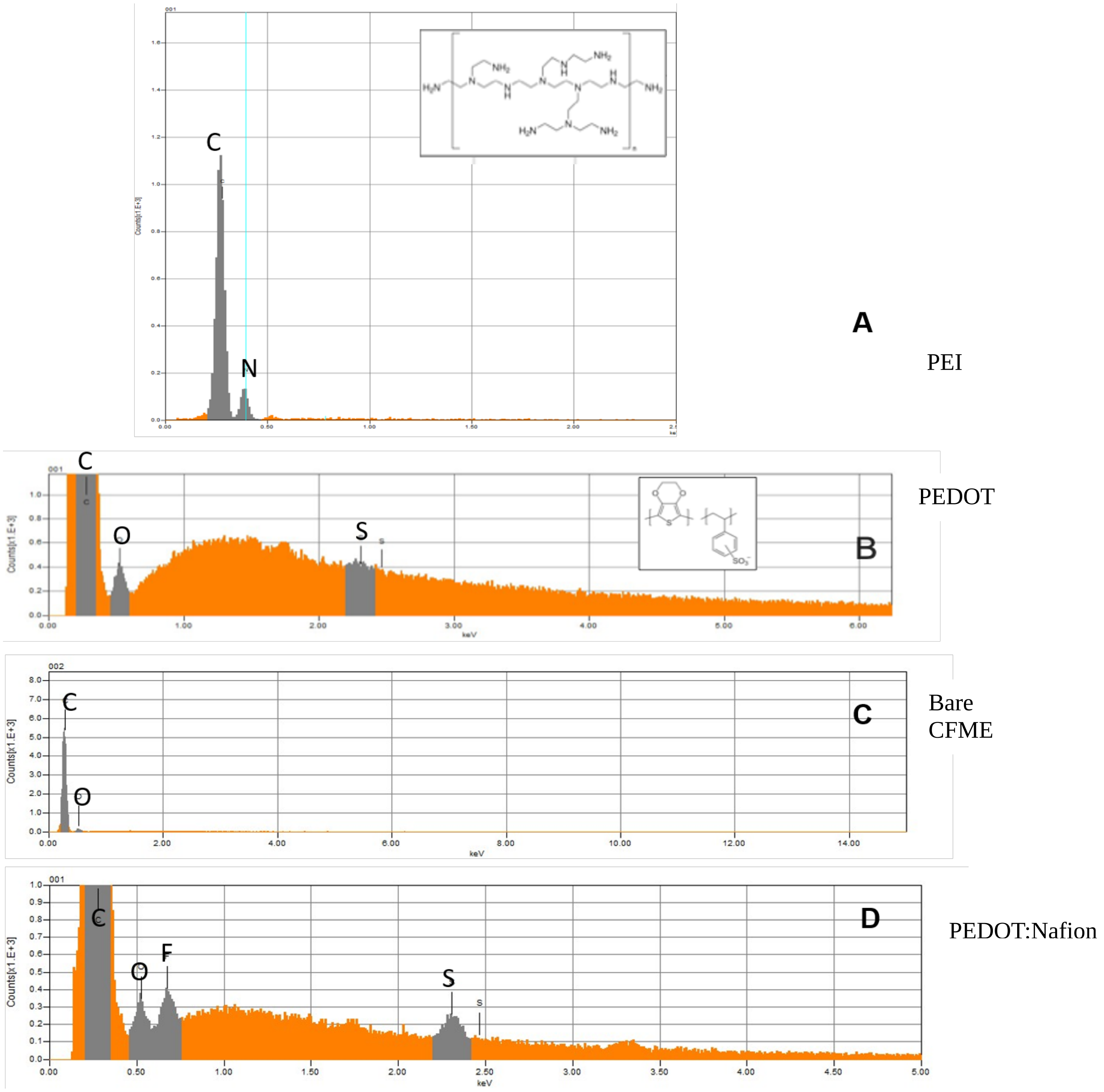

Figure S1. Energy-dispersive X-Ray (EDS/EDX) analysis of (A) a carbon fiber coated with PEI polymer confirming the presence of nitrogen (B) a carbon fiber coated with PEDOT-PEI polymer confirming the presence of oxygen and sulfur (C) a bare carbon fiber and (D) a carbon fiber coated with PEDOT-Nafion polymer.

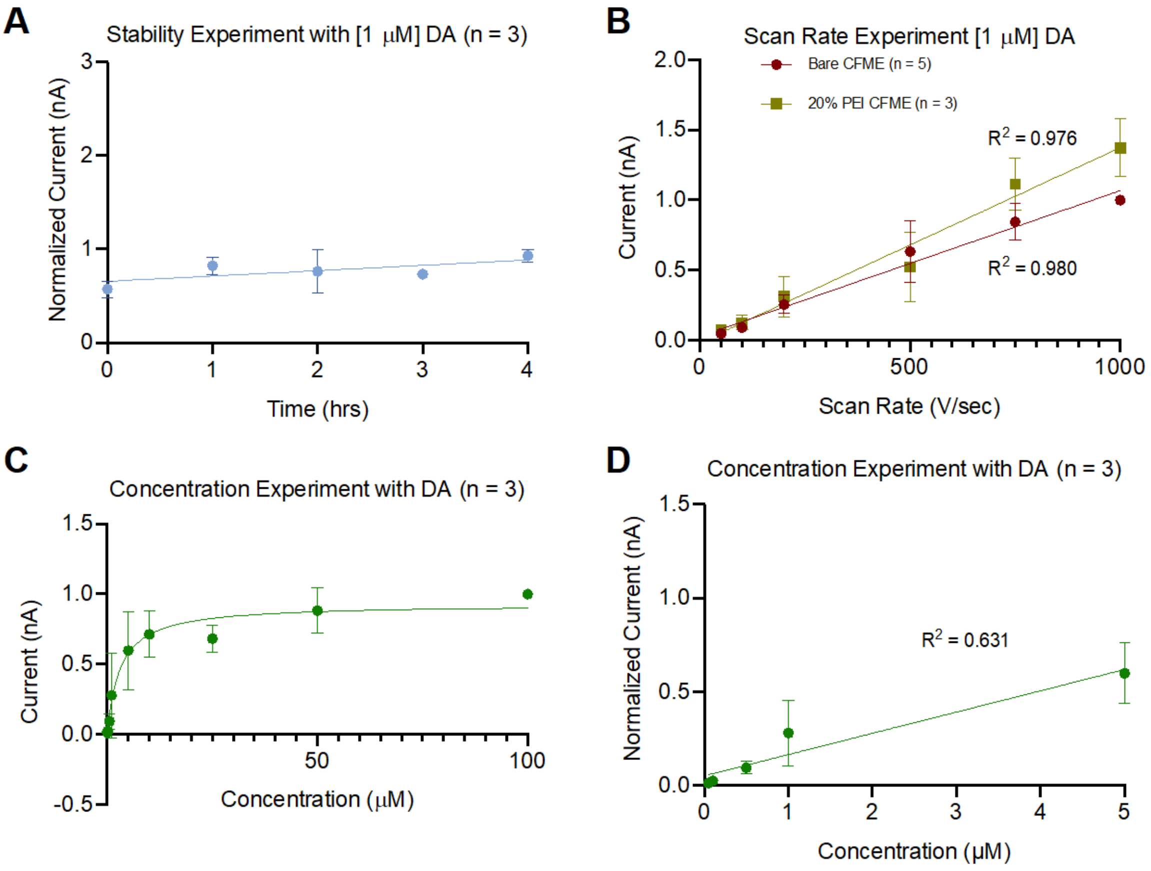

Figure S2. Adsorption Control Experiments with Dopamine (Bare and PEI CFMEs)

(A) Stability test shows a stability towards DA detection (peak oxidative current) for at least four hours (n = 3). (B) Adsorption control testing for bare and PEI polymer coated CFMEs. The peak oxidative current for DA (1 µM) are linear with respect to scan rate (50-1000 V/s), suggesting adsorption control to the surface of the bare and polymer coated CFMEs. The PEI coated CFMEs shows an enhanced sensitivity. R^2 = 0.980 and 0.976, respectively. (C) Concentration testing of bare CFMEs. The peak oxidative currents for DA are saturated at the surface of the electrode, which blocks further adsorption. R^2 = 0.970 (n = 3). (D) The peak oxidative currents for DA are linear with respect to concentration from 100 nM to 10 µM.

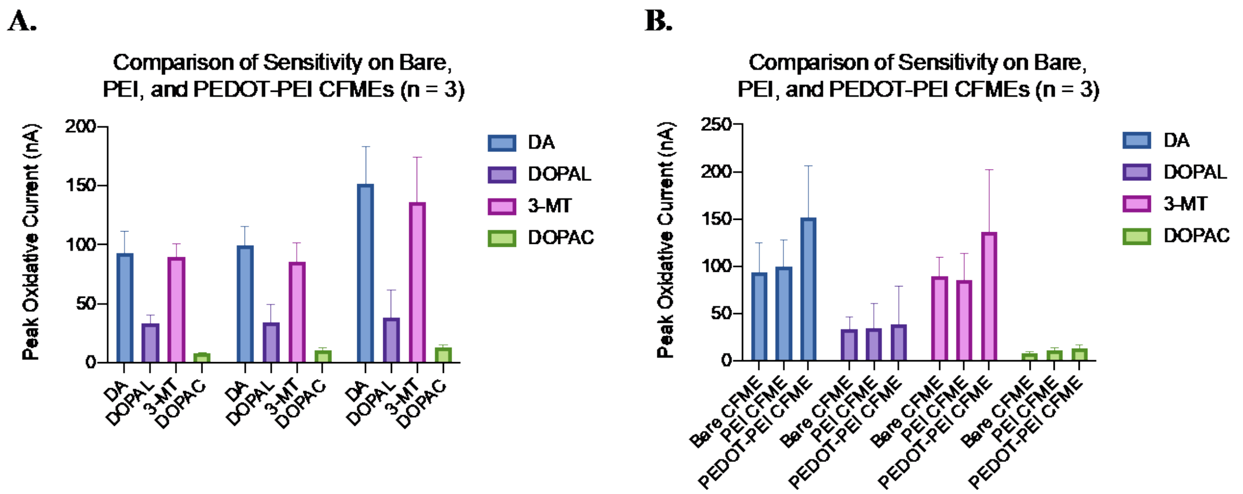

Figure S3. Effect of PEI and PEDOT-PEI polymer coatings on the detection sensitivity of dopamine (DA), 3-methoxytyramine (3-MT), 3,4-Dihydroxyphenylacetaldehyde (DOPAL), and 3,4-Dihydroxyphenylacetic acid (DOPAC). The currents were obtained from the raw data and averaged for each neurotransmitter (n = 3).

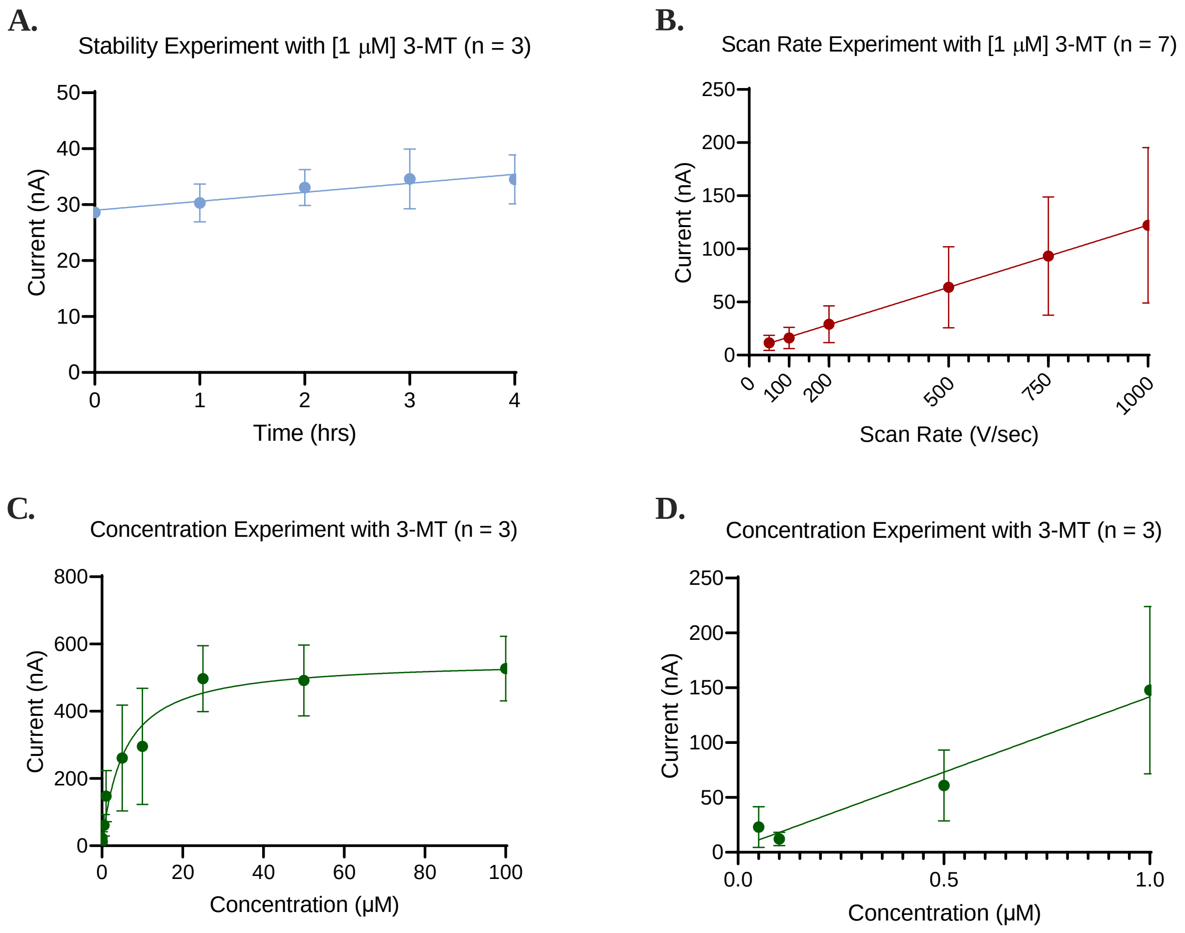

Figure S4. Adsorption control testing of CFMEs using raw data. (A) The electrode displays a stability towards 3-methoxytyramine (3-MT) detection (peak oxidative current) for at least 4 hours. (n = 3). (B) Adsorption control testing for 3-MT (1 µM) showing linear relationship between peak oxidative current and scan rate (50-1000 V/s, n = 7). (C) The peak oxidative currents of the cyclic voltammograms (CV) for 3-MT showing asymptotic curve with respect to the concentration (n = 3). (D) Concentration experiment showing a linear relationship between 3-MT concentration (from 100 nM to 1 µM) and peak oxidative current (n = 3).

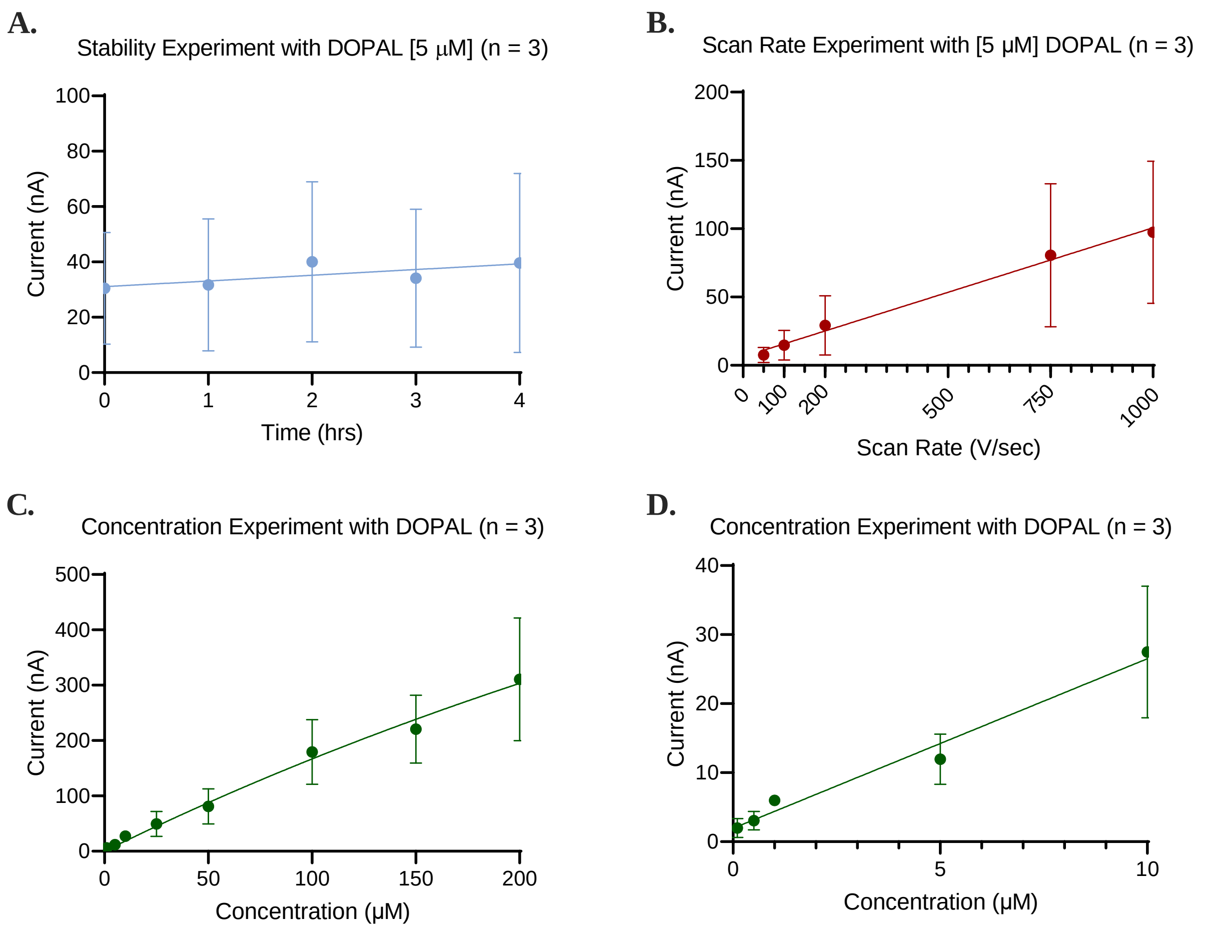

Figure S5. Adsorption control testing of CFMEs with raw data. (A) The electrode displays a stability towards DOPAL detection (peak oxidative current) for at least 4 hours (n = 3). (B) Adsorption control testing for DOPAL (5 µM) showing a linear relationship between scan rate (50-1000 V/s) and peak oxidative current (n = 3). (C) The peak oxidative currents for the cyclic voltammograms (CV) for DOPAL with respect to the concentration (n = 3). (D) Concentration showing a linear relationship between DOPAL concentration (100 nM to 10 µM) and peak oxidative current (n = 4).

Relative Concentration [µM]		
**Ratio**	**DA**	**3-MT**
**100:1**	5	0.05
**10:1**	5	0.5
**1:1**	2.5	2.5
**1:10**	0.5	5
**1:50**	0.08	4
**1:100**	0.05	5

Relative Concentrations [µM]		
**Ratio**	**DA**	**DOPAL**
**100:1**	5	0.05
**10:1**	5	0.5
**1:1**	4	4
**1:10**	1.4	14
**1:50**	0.4	20.0
**1:100**	0.2	20.0

Table S1. The co-detection selectivity was evaluated using DA versus both 3-MT and DOPAL, respectively, for both the bare and PEDOT-PEI coated CFMEs with selectivity defined as the ratio of sensitivity to the analyte. The 1:1 ratio for DA versus 3-MT and DA versus DOPAL were prepared to have the same relative concentrations. DA's concentration remained the same while the 3-MT and DOPAL concentrations were lowered for the 10:1 and 100:1 ratio, respectively. Moreover, DA's concentration was decreased while the 3-MT and DOPAL concentrations remained constant for the 1:10, 1:50, and 1:100 ratio. These ratios refer to the ratios used in Figures 5 and 6.
